# Microbial Spectrum of Keratitis at a Rural Tertiary Care Teaching Hospital

**DOI:** 10.7759/cureus.55055

**Published:** 2024-02-27

**Authors:** Bipin Chandra Bhagath, Subbarama Prasad, Arvind Natarajan

**Affiliations:** 1 Microbiology, Sri Devaraj Urs Academy of Higher Education and Research, Kolar, IND

**Keywords:** demographic characteristics, climate weather, occupation, aetiological agents, microbial keratitis

## Abstract

Introduction: Microbial keratitis poses a significant threat to vision and is a common ocular infection. Its causative agents encompass a wide spectrum, including bacteria, fungi, viruses, and parasites. The microbiological profile of microbial keratitis is influenced by factors such as patient demographics, geographical location, climate, and occupational hazards and evolves over time.

Methodology: Corneal scrapings were collected from 75 patients with a provisional diagnosis of microbial keratitis. The samples were processed in the microbiology laboratory, and the bacterial and fungal growth isolated in the study were identified according to standard procedures.

Results: Among the 75 patients, 48 (64%) were male and 27 (36%) were female. Corneal ulceration was found in individuals of all age groups, with the highest prevalence of 77.33% (58/75) observed in the 21-60 age range. Farmers exhibited a higher susceptibility, constituting 66.67% (50/75) of the cases. The study noted a higher occurrence of keratitis from November to February, accounting for 69.33% (52/75). Microbial etiology was identified in 25.33% (19/75) of scrapings, with fungi accounting for 68.42% (13/19) and bacteria for 31.57% (6/19). The prevalent fungal species included *Fusarium *(7/13, 53.84%), *Aspergillus *(3/13, 23.07%), *Colletotrichum *(2/13, 15.38%), and *Curvularia *​​​​​​​(1/13, 7.69%). Bacterial isolates featured *Streptococcus pneumoniae* (5/6, 83.33%) and *Klebsiella pneumoniae *(1/6, 16.66%).

Conclusions: This study underscores the importance of regularly updating local microbial profiles and understanding antimicrobial resistance patterns. Such updates are critical for informed decision-making in selecting optimal topical treatments for microbial keratitis.

## Introduction

Microbial keratitis significantly threatens ocular health, posing a common and potentially vision-endangering infection. Microbial keratitis can be caused by diverse pathogens viz bacteria, fungi, viruses, and parasites [1]. These microorganisms cause corneal damage through direct invasion by utilizing their virulence factors or triggering the host's immune response [2]. Various risk factors, such as contact lens usage, ocular injuries, dry eye, epithelial defects, systemic illnesses, and immunosuppression, contribute to the development of microbial keratitis. Left untreated, the condition can progress to corneal ulceration and scarring, leading to blindness [3]. The microbiological profile of microbial keratitis is influenced by diverse factors, including patient demographics, geographic location, climate, and occupational risks, and tends to evolve over time [4].

Hence, understanding the local epidemiological features, predisposing factors, and causative agents specific to a region is crucial for prompt identification, proper management, and prevention of this infection [5]. The objective of this study was to investigate the sociodemographic aspects and determine the microbiological profile of microbial keratitis. The findings aim to provide explicit data for ophthalmologists and policymakers, enabling the formulation of guidelines for the effective management of microbial keratitis.

## Materials and methods

The study design entailed a prospective observational approach focused on 75 patients suspected of having microbial keratitis who sought medical attention at either the Ophthalmology Outpatient clinic or the emergency department. All patients, regardless of gender or age, who were provisionally diagnosed with Microbial keratitis, were included. Those with certain ulcer types like healing ulcers, marginal ulcers, perforated ulcers, typical viral ulcers, sterile neurotropic ulcers, or ulcers linked to autoimmune conditions were excluded. Also, patients who declined to consent to sample collection were not included.

Data collection involved gathering sociodemographic details, associated risk factors, treatment history, and clinical observations using a predefined proforma. Informed consent was obtained from all patients. Corneal scrapings were performed by ophthalmologists. In aseptic conditions, a sterile 26-gauge needle was used after the application of 4% lignocaine (lidocaine) to carefully scrape the base of the ulcer, ensuring access to sufficient infective material potentially located deep within or at the edges of the ulcer [6]. The collection involved moderately steady, unidirectional strokes under a slit lamp light. Microscopic examination of corneal scrapings included Gram stain, 10% potassium hydroxide (KOH) mount, and saline mount. Aseptic inoculation of scrapings was done on chocolate agar, blood agar, MacConkey's agar, Sabouraud's dextrose agar (SDA), potato dextrose agar, thioglycolate, and brain heart infusion broths. The culture media were incubated appropriately for growing bacterial and fungal pathogens.

Identification of bacterial and fungal isolates adhered to standard procedures [7]. Fungal isolates were characterized by colony morphology on specific culture media, pigmentation, growth rates, and microscopic features, including micrometry on lacto phenol cotton blue mount and slide culture. Microbial cultures were considered significant if the same organism grew on two or more solid media and if smear and culture results correlated with clinical findings. The antibiotic sensitivity of bacterial isolates was determined using the modified Kirby-Bauer disc diffusion method [8]. Minimum inhibitory concentration levels of Penicillin against pneumococcal isolates were identified using the Vitek 2 System. Statistical analysis involved the chi-square test to compare proportions, with a significance level set at *P* < 0.05.

## Results

The epidemiological characteristics of patients with corneal ulcers are detailed in Table 1.

**Table 1:** Epidemiological characteristics of patients with corneal ulcers. ^*^Splinter/dust exposure occupations include welders (4), mechanics (3), stonecutters (2), iron bar benders (1), carpenters (1), and drivers (1).

Demographics (*n *= 75)	Indicators	n	%
Age (years)	1-20	6	8.00
	21-40	38	50.66
	41-60	20	26.67
	61-80	11	14.66
Gender	Male	48	64.00
	Female	27	36.00
Occupation	Farmers	50	66.67
	Splinter/dust exposure occupations*	12	16.00
	Housewives	8	10.67
	Students	5	6.67
Seasonal variation	Mar-June	12	16.00
	July-Oct	11	14.67
	Nov-Feb	52	69.33

The clinical characteristics of corneal ulcers are illustrated in Table 2.

**Table 2:** Clinical characteristics of corneal ulcer.

Clinical characteristics	*n* (%)
Size of the ulcer (mm)	
≤4	48 (64)
>4	27 (36)
Location of ulcer	
Paracentral	55 (73.33)
Peripheral	08 (10.66)
Central	12 (16)
Presentation interval	
≤4 days	42 (56)
﹥4 days	33 (44)
Hypopyon	
Present	13 (17.33)

Microbial etiology was identified in 25.33% (19/75) of cases, with fungi accounting for 68.42% (13 cases) and bacteria for 31.57% (6 cases), as illustrated in Table 3.

**Table 3:** Etiological agents of corneal ulcers.

Isolates	Number of isolates, *n*	%
Fungal isolates (n = 13)		
Fusarium semitectum	3	23.08
Fusarium solani	2	15.38
Fusarium dimerum	2	15.38
Aspergillus flavus	2	15.38
Aspergillus niger	1	7.69
Colletotrichum gloeosporioides	2	15.38
Curvularia lunata	1	7.69
Bacterial isolates (n = 6)		
Streptococcus pneumoniae	5	83.33
Klebsiella pneumoniae	1	16.67

Antibiotic susceptibility results

All five strains of *Streptococcus pneumoniae* were sensitive to penicillin, erythromycin, gentamicin, chloramphenicol, tetracycline, ciprofloxacin, linezolid and vancomycin. *Klebsiella pneumoniae* was also sensitive to gentamicin, amikacin, cefuroxime, cefixime, ciprofloxacin, chloramphenicol, tetracycline, co-trimoxazole, and amoxicillin-clavulanate but resistant to ampicillin.

**Figure 1:**
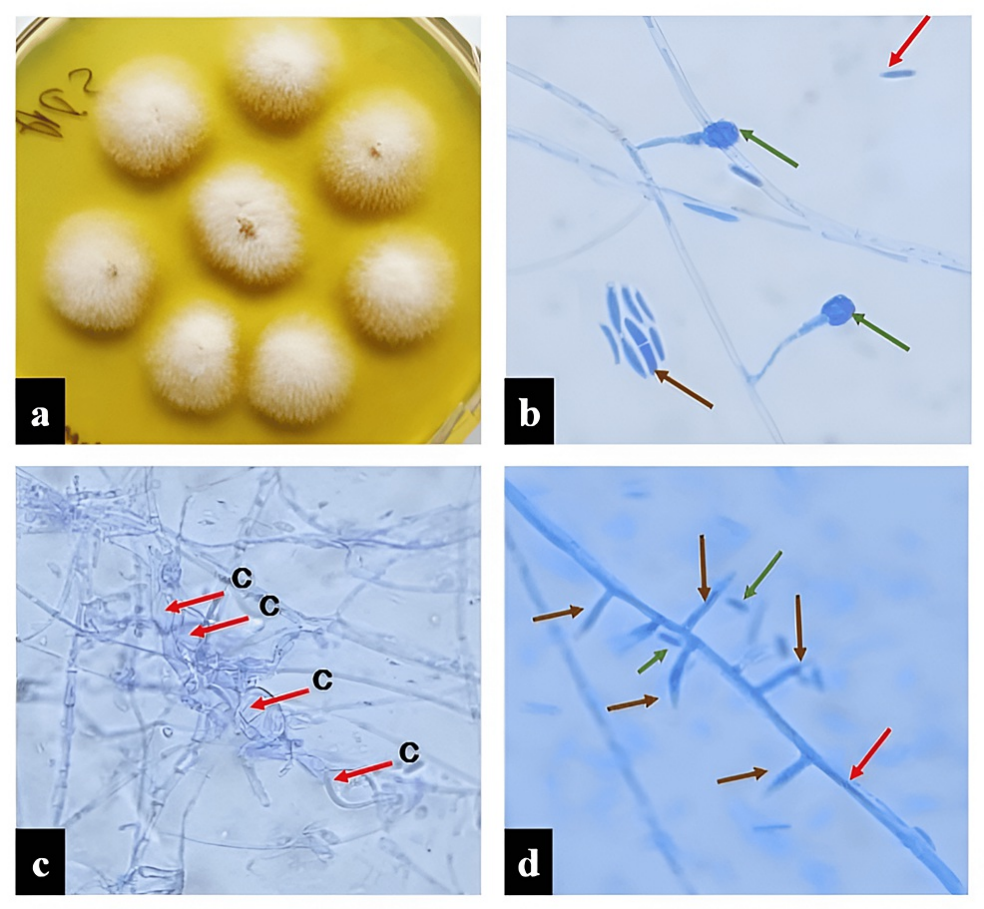
Fusarium semitectum. (a) Cottony, white colonies on SDA after seven days of incubation. (b) LPCB mount showing macroconidia (brown arrow), microconidia (red arrow), and chlamydospores (green arrow). (c) Terminal to intercalary chlamydospores (red arrow) in chains. (d) Short, lateral monophilades (brown arrow) with microconidia (green arrow) and with conidiophore (red arrow) (b-d: at x40). SDA, Saborauds dextrose agar; LPCB, lactophenol cotton blue

**Figure 2:**
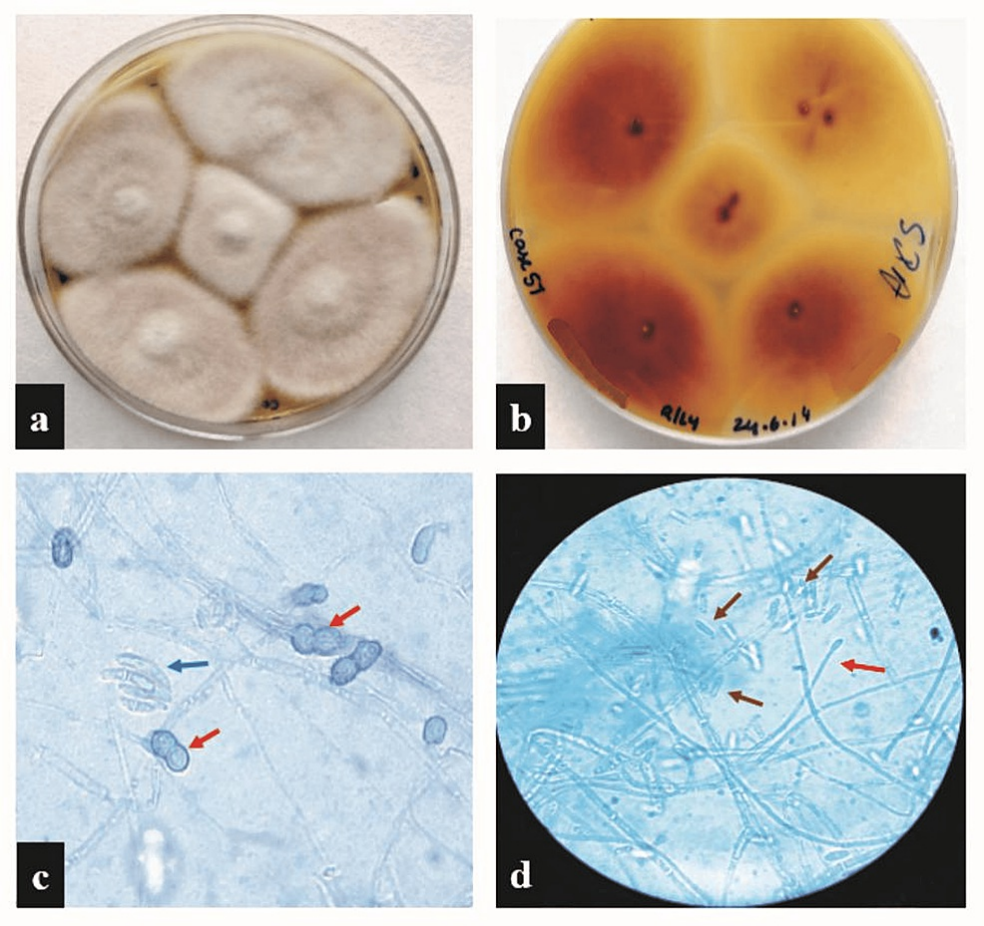
Fusarium solani. (a) Grayish white floccose irregular colonies on SDA after five days of incubation. (b) Reverse showing umber color. (c) LPCB mount showing abundant macroconidia (blue arrow) and rough-walled chlamydospores (red arrow). (d) Long monophialide (red arrow) along with abundant microconidia (brown arrows). (c-d) At x40. SDA, Saborauds dextrose agar; LPCB, lactophenol cotton blue

**Figure 3:**
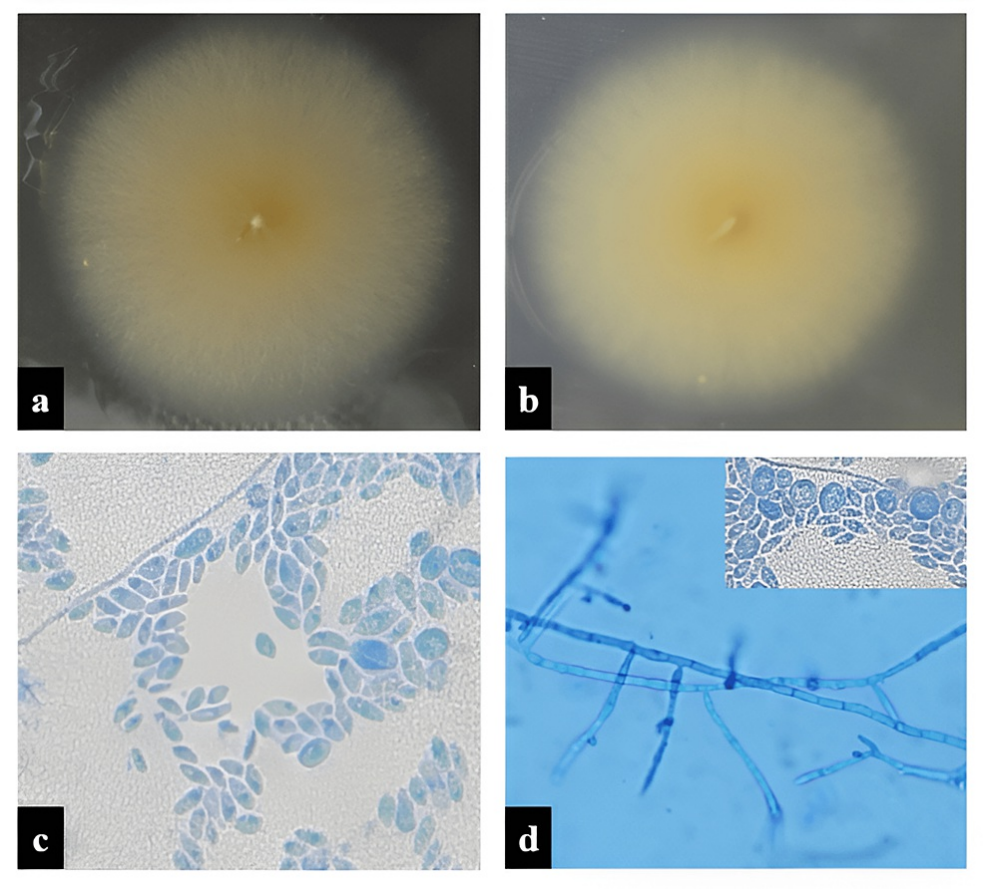
Fusarium dimerum. (a) Floccose colonies on SDA after seven days of incubation. (b) The colony shows a umber color on the reverse. (c) LPCB mount showing micro and macroconidia. (d) Monoto polyphialides arising from prostrate mycelium. Presence of subglobose to globose chlamydospores in inset view (c-d: at x40). SDA, Saborauds dextrose agar; LPCB, lactophenol cotton blue

**Figure 4:**
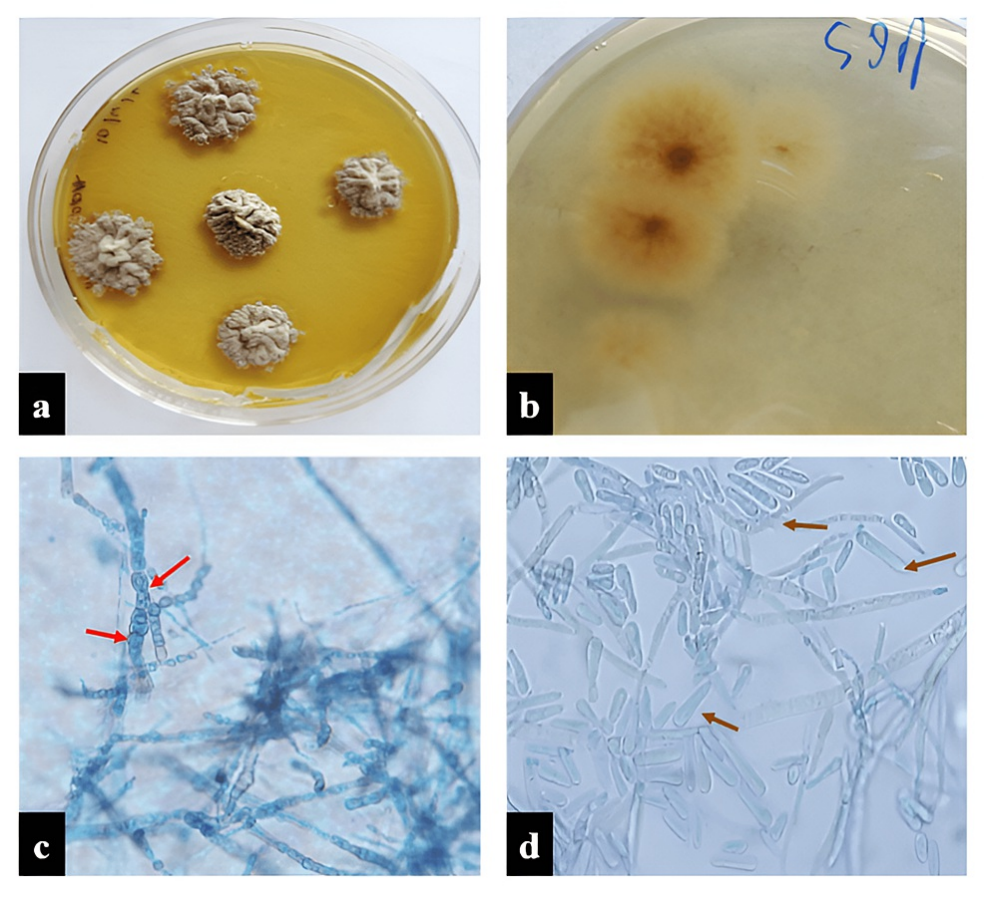
Colletotrichum gloeosporioides. (a) Gray colonies on SDA after seven days of incubation. (b) A slate brown reverse colony color. (c) LPCB mount showing appressoria (red arrow). (d) Abundant, hyaline straight or cylindrical conidia (c-d: at x40). SDA, Saborauds dextrose agar; LPCB, lactophenol cotton blue

The primary fungal species isolated in this study were *Fusarium *species, specifically *Fusarium semitectum*, *Fusarium solani*, and *Fusarium dimerum*, prominently depicted in Figures 1-3. Following *Fusarium*, *Aspergillus* species (*Aspergillus flavus* and *Aspergillus niger*); *Colletotrichum* species (*Colletotrichum gloeosporioides*), as depicted in Figure 4; and *Curvularia* species were also observed.

The initial treatment regimen involved empirical topical antibiotics, both with and without topical antifungal therapy. In severe cases, systemic antibiotics and systemic antifungal agents were introduced as supplementary measures. In our cases, the topical antibiotics employed included fortified tobramycin, gatifloxacin, and moxifloxacin. Additionally, the topical antifungals comprised natamycin, voriconazole, and fluconazole. In this study, 38 (50%) patients received empirical coverage with two topical antibiotics and two topical antifungals. Twenty-one (28%) patients initiated treatment solely with topical antifungals, while 16 (21.3%) patients started with only topical antibiotics. Additionally, 14 (18.66%) cases were administered systemic antibiotics, and 11 (14.66%) cases received systemic antifungal therapy during the treatment period. Six (7%) patients underwent a switch from antibiotics to antifungals based on microbiology reports and clinical characteristics. The duration of treatment varied on a case-by-case basis; however, it typically spanned approximately three to four weeks for bacterial keratitis and six to eight weeks for fungal keratitis.

Thirteen patients diagnosed with a fungal etiology were monitored over two weeks, during which they displayed signs of symptom relief and improved visual acuity. Among the six patients identified with a bacterial etiology, five experienced symptom improvement and enhanced visual acuity, while one patient was lost to follow-up. In our study, the majority of cases (48, 64%) achieved complete healing, attributed to smaller ulcer sizes and the sensitivity of most strains to treatment. Only two cases necessitated keratoplasty. Initially, presenting visual acuity was <6/60 in 29 cases and ≥6/60 in 46 cases. After treatment completion, there was a significant improvement in visual acuity, with <6/60 in 14 cases and ≥6/60 in 61 cases.

## Discussion

Microbial keratitis stands as a leading cause of preventable blindness globally acknowledged by the World Health Organization (WHO) as one of the neglected tropical diseases [9,10]. In our study, corneal ulceration was evident across all age groups, with the highest prevalence of 77.33% (58/75) observed in individuals aged 21-60 years. This aligns with findings from Das and Konar, Chauhan et al., and Cameron et al., suggesting the active engagement of this age group in outdoor activities and work [2,11,12]. Conversely, Suja et al. and Keay et al. reported a higher incidence among the elderly, attributing it to factors such as poor cell-mediated immunity, malnutrition, and comorbidities [13,14].

The male predominance of 64% (48/75) observed in our study, consistent with several other studies, can be attributed to the higher involvement of males in outdoor activities, making them more prone to injuries [4,11,13,14]. However, studies by Hoffman and Suwal et al. reported a female preponderance, possibly linked to females' higher employability in agricultural work [6,15].

Occupational and environmental factors play a crucial role in the array of microbial keratitis [16]. Our study highlighted a notable predominance among farmers at 66.67% (50/75), in line with Tewari et al., Suwal et al., and Srinivasan et al. [4,15,17]. This association could be linked to vegetative trauma in cultivation fields. Additionally, our study unveiled housewives as a significant risk group, engaging in activities like winnowing, dusting, and wood chipping in developing countries. Another interesting finding in our study is that individuals employed in occupations exposed to dust and splinters, along with housewives, also featured prominently among those who presented with corneal ulcers [18].

Contrary to the findings of Bharathi et al., who associated fungal keratitis with hot, windy environments typical of tropical zones, our study observed a higher incidence of 69.33% (52/75) cases between November and February, correlating with winter months and harvesting activities [16,19]. The difference underscores the regional variations in keratitis patterns.

In our study, many cases of microbial keratitis were found to be sterile, likely attributable to factors such as minimal microbial presence in samples, the body's immune response, environmental influences, and prior administration of antimicrobial treatment at local clinics before hospital admission. Despite the cause, monitoring for negative growth remains vital in microbial keratitis management to ensure effective control and deter further advancement, which could lead to complications like corneal scarring or vision impairment. Regular follow-up is essential for evaluating treatment response and determining subsequent management steps. Microbial etiology was identified in 25.33% (19/75) of cases, predominantly fungal (13/19, 68.42%) followed by bacterial (6/19, 31.57%) cases [4]. *S. pneumoniae *emerged as the dominant bacterial isolate, aligning with Bharathi et al.'s findings [20]. In contrast, most studies typically report *Staphylococcus aureus* as predominant, preceded by coagulase-negative *Staphylococcus* [21]. Our study did not reveal multi-drug-resistant strains among bacterial isolates. This is reassuring and suggests a cautious use of antibiotics in the studied population.

*Fusarium *species took precedence in our study, particularly *F. semitectum, F. solani,* and *F. dimerum.* Regional variations were evident, with studies by Roy et al. and Jisha et al. reporting similar results [22,23], while Alkatan et al. and Abouzeid et al. identified *Aspergillus* as the preponderant fungus [24,25]. The fungi isolated from corneal ulcers in India are reported to show regional variation. As per Manikandan et al., *Aspergillus* species predominate in northern India, whereas *Fusarium* species predominate in the southern part of the country [26].

The spores of *Aspergillus* species are omnipresent in the surroundings, whereas *Fusarium *species are frequent plant pathogens and can get admittance in case of any injury by vegetative substance. Thomas and Kaliamurthy observed that keratitis caused by *Fusarium *species has a much more invasive nature and is a relatively inferior response to management than *Aspergillus *species [27].

Out of the seven isolates of *Fusarium*, three could be morphologically identified as *F. semitectum*, two as *F. solani*, and two as *F. dimerum*. While *F. solani* is frequently identified in corneal ulcers in India, the isolation of *F. semitectum* and *F. dimerum* is infrequent [5]. *F. semitectum *is a well-known plant pathogen associated with post-harvest fruit rot diseases of tomato [28]. The abundance of *Fusarium* species in our study region could be linked to its role as a plant pathogen and the prevalent tomato cultivation in the area. In our study, we identified two cases of microbial keratitis caused by *C. gloeosporioides*. Keratitis caused by *Colletotrichum* species is rare, and only a few studies have reported it. So far, only five *Colletotrichum* species are related to rare human eye infections: *Colletotrichum* *dematium, C. gloeosporioides, Colletotrichum crassipes, Colletotrichum* *graminicola, and Colletotrichum coccodes *[29]. The diversity in preponderant fungal isolates may result from climatic and environmental variations across regions.

Limitations of our study include its single-center design. Future multi-centric studies would provide a broader perspective. Furthermore, our study did not identify anaerobes, viral, and amoebic agents contributing to microbial keratitis, indicating the necessity for comprehensive multi-centric investigations integrating molecular diagnostics such as real-time polymerase chain reaction (RT-PCR), confocal microscopy, cell lines, and specialized culture media to encompass all infectious agents involved.

## Conclusions

Microbial keratitis remains a prevalent cause of visual impairment, particularly in developing nations like India. The diverse causative agents, encompassing both bacteria and fungi, underscore the importance of recognizing the variability in microbiological profiles across different geographical regions and climatic conditions.

The key takeaway from our study is the critical need for ongoing local updates on microbial profiles and antimicrobial resistance patterns. This information is indispensable for guiding ophthalmologists in selecting the most appropriate topical treatments for microbial keratitis.

As the epidemiological landscape evolves, these updates ensure that therapeutic strategies align with the prevalent microbial strains. This ultimately contributes to more effective and targeted management of this sight-threatening condition.
